# Genetic Particle Swarm Optimization–Based Feature Selection for Very-High-Resolution Remotely Sensed Imagery Object Change Detection

**DOI:** 10.3390/s16081204

**Published:** 2016-07-30

**Authors:** Qiang Chen, Yunhao Chen, Weiguo Jiang

**Affiliations:** 1 State Key Laboratory of Earth Surface Processes and Resource Ecology, Beijing Normal University, Beijing 100875, China; chenqiang@mail.bnu.edu.cn; 2 College of Resources Science and Technology, Beijing Normal University, Beijing 100875, China; 3 Academy of Disaster Reduction and Emergency Management Ministry of Civil and Ministry of Education, Beijing Normal University, Beijing 100875, China

**Keywords:** feature selection, particle swarm optimization, change detection, remote sensing

## Abstract

In the field of multiple features Object-Based Change Detection (OBCD) for very-high-resolution remotely sensed images, image objects have abundant features and feature selection affects the precision and efficiency of OBCD. Through object-based image analysis, this paper proposes a Genetic Particle Swarm Optimization (GPSO)-based feature selection algorithm to solve the optimization problem of feature selection in multiple features OBCD. We select the Ratio of Mean to Variance (RMV) as the fitness function of GPSO, and apply the proposed algorithm to the object-based hybrid multivariate alternative detection model. Two experiment cases on Worldview-2/3 images confirm that GPSO can significantly improve the speed of convergence, and effectively avoid the problem of premature convergence, relative to other feature selection algorithms. According to the accuracy evaluation of OBCD, GPSO is superior at overall accuracy (84.17% and 83.59%) and Kappa coefficient (0.6771 and 0.6314) than other algorithms. Moreover, the sensitivity analysis results show that the proposed algorithm is not easily influenced by the initial parameters, but the number of features to be selected and the size of the particle swarm would affect the algorithm. The comparison experiment results reveal that RMV is more suitable than other functions as the fitness function of GPSO-based feature selection algorithm.

## 1. Introduction

Object change detection in Very-High-Resolution (VHR) remotely sensed imagery has become a hot topic in the field of remotely sensed imagery analysis, and object-oriented image analysis has been the primary way to solve the “salt and pepper” problem [1], which commonly occurred in pixel-based image analysis. In the field of object-based change detection (OBCD), VHR imagery is usually segmented to several objects and the image objects are regarded as the basic processing units. The main difference between pixel-based change detection and object-based change detection is that image objects have more feature information, so multi-feature image analysis can identify more change information for VHR remotely sensed imagery [2,3].

Feature selection selects a subset of relevant features from the available features to improve change detection performance relative to using single features. Existing feature selection algorithms can be broadly classified into two categories: filter approaches and wrapper approaches [4]. Wrappers achieve better results than filters, because wrappers include a learning algorithm as part of the evaluation function. There are two components of a feature selection algorithm: the search algorithm and the evaluation function [5]. Common methods of feature selection are principal component analysis [6], Sequential Forward Selection (SFS) [7], Sequential Float Forward Selection (SFFS) [8] and Backtracking Search Optimization Algorithm (BSO) [9]. It is difficult to implement a global search in the high-dimensional feature space for these algorithms. Further, the search process is separate from the evaluation process, so it is easy to fall into the trap of local convergence. Subsequently, Evolutionary Computation (EC) algorithms have been used for the feature selection problem, such as simulated annealing [10], Genetic Algorithms (GA) [11,12], Differential Evolution algorithm (DE) [13] and swarm intelligent algorithms, in particular Cuckoo Search (CS) [14,15], ant Colony Optimization (CO) [16] and Particle Swarm Optimization (PSO) [17]. These algorithms have the advantages of high efficiency, high speed and low cost, but they also have random components and some results are hard to reproduce in experiments.

As a typical example of EC algorithms, PSO has a high search ability and is computationally less expensive than some other EC algorithms, so it has been used as an effective technique in feature selection. Starting with a random solution, PSO searches for the optimum solution in the feature space according to some mechanism, and the search space can be expanded to the whole problem space without incurring more cost. That is, PSO is an adaptive probabilistic search algorithm, and only the objective function rather than gradient information is needed to evaluate the fitness of solutions. Moreover, PSO is an easily implemented algorithm, has less adjustable parameters and is also computationally inexpensive in both speed and memory requirements [18].

The genetic particle swarm optimization (GPSO) algorithm integrates PSO with GA to improve solution speed and global search ability, and has been widely used in optimal selection problems [18,19,20,21,22]. GPSO has some advantages such as optimization ability of fast approaching to the optimal solution, simple parameters setup and high global search ability to avoid the local optimal problem. But the drawbacks of GPSO mainly include that it easily falls into the local optimal solution in late iteration, and the different parameters setup, such as swarm scale, would affect the efficiency and cost of algorithm [18]. Nazir proposed an efficient gender classification technique for real-world facial images based on PSO and GA selecting the most import features set for representing gender [19]. Inbarani proposed a supervised feature selection method based on hybridization of PSO and applied this approach to diseases diagnosis [20]. Yang proposed a novel feature selection and classification method for hyperspectral images by combining PSO with SVM, and the results indicate that this hybrid method has higher classification accuracy and effectively extracts optimal bands [21]. Chuang et al. [22] applied the so-called catfish effect to PSO for feature selection, claiming that the catfish particles help PSO avoid premature convergence and lead to better results than sequential GA, SFS, BSO and other methods. Additionally, GPSO has also been applied in the field of remotely sensed imagery analysis, such as image segmentation [23], image classification [24,25] and hyperspectral band selection [26]. Up to now, several PSO-based feature selection methods have been proposed in the literature [4,18,25,27,28]. However, GPSO-based feature selection has seldom been used in remotely sensed image change detection.

Fitness functions are an important part of GPSO-based feature selection algorithms, and they are used to evaluate the selected feature subset during the search process, by comparing the current solution with established evaluation criterion for particle updates and termination of the procedure. Fitness functions are commonly built based on correlation, distance metrics and information gain. Furthermore, the classification error rate of a classifier also has been used to build the fitness function in the wrapper feature selection method. It is worth noting that the fitness function used for GPSO should be related to the aim of the research.

Existing studies on OBCD for VHR remotely sensed imagery base feature selection primarily on expert knowledge or experimental data, both of which have a low efficiency rate and a low precision rate [29]. This paper proposes a GPSO feature selection algorithm to be utilized in OBCD by using object-based hybrid multivariate alternative detection (OB-HMAD) model, and analyzes the fitness convergence and OBCD accuracy of the algorithms using the ratio of mean to variance (RMV) as the fitness function. Additionally, we discuss the sensitivity of the number of features to be selected and the scale of the particle swarm to the precision and efficiency of the algorithm, and analyze the reliability of RMV by comparing with other fitness functions. Therefore, our paper is organized into five sections. Section 2 presents the theoretical constructions behind our proposed GPSO-based feature selection approach using a RMV fitness function, and illustrates how our technique can be used for multiple features OBCD. Analysis of our results obtained for two experimental cases are reported in the Section 3, sensitivity of GPSO-based feature selection algorithm has been discussed in the Section 4, while Section 5 outlines our conclusions.

## 2. Methodology

### 2.1. Genetic Particle Swarm Optimization

PSO uses an information-sharing mechanism that allows individuals to learn from each other to promote the development of the entire swarm. It has excellent global search ability even in high-dimensional solution spaces. In PSO, possible solutions are called particles, and each particle *i* comprises three parts: *x_i_*, the current position of the particle; *v_i_*, the current velocity of the particle, which also denotes the recursive solution update; and *pbest_i_*, the personal best position of the particle which is the best local solution. The solution space is searched by starting from particles randomly distributed as in a swarm.

Assume *M* features are to be selected, and let a particle *x_i_* (of size *M* × 1) denote the selected feature indices, and *v_i_* denote the update for selected feature indices. Then the particle swarm is composed of *N* particles (*N* is the size of particle swarm), and every particle has a position vector *x_i_* to indicate its position and a velocity vector *v_i_* to indicate its flying direction and speed. As the particle flies in the solution space, it stores the best local solution *pbest_i_* and the best global solution *gbest_i_.* Here, possible solutions are called particles, and recursive solution update is called velocity. Initially, the particles are distributed randomly and updated depending on the best local solution *pbest_i_* and global solution *gbest_i_*. The algorithm then searches for the optimal solution by updating the position and the velocity of each particle according to the following equations:

(1) $$\nu_{i}\left(t+1\right)=\omega \nu_{i}\left(t\right)+c_{1}r_{1}\left(pbest_{i}\left(t\right)-x_{i}\left(t\right)\right)+c_{2}r_{2}\left(gbest_{i}\left(t\right)-x_{i}\left(t\right)\right)$$

(2) $$x_{i}\left(t+1\right)=x_{i}\left(t\right)+\nu_{i}\left(t+1\right)$$

where *i* = 1,2,…,*n*, *N* is the total number of particles in the swarm, *r*_1_ and *r*_2_ are random numbers chosen uniformly from (0,1), *c_1_* and *c_2_* are learning factors (*c_1_* denotes the preference for the particle’s own experience, and *c_2_* denotes the preference for the experience of the group), *t* is the number of iteration, and *ω* is the inertia weight factor that controls the impact of the previous velocity *v_i_* which provides improved convergence performance in various applications [30]. Because feature indices are discrete values, rounding off the solutions to adapt the continuous PSO to a discrete form is necessary.

Adapted from the GA, a crossover operator is used in PSO to improve the global searching capability and to avoid running into local optimum. After updating the position vector *x_i_* and the velocity vector *v_i_* of the particle using Equations (1) and (2), the algorithm calculates a crossover with two particles, as follows:

(3) $$child_{1}\left(x_{i}\right)=\omega_{i}\times parent_{1}\left(x_{i}\right)+\left(1-\omega_{i}\right)\times parent_{2}\left(x_{i}\right)$$

(4) $$child_{2}\left(x_{i}\right)=\omega_{i}\times parent_{2}\left(x_{i}\right)+\left(1-\omega_{i}\right)\times parent_{1}\left(x_{i}\right)$$

(5) $$child_{1}\left(\nu_{i}\right)=\frac{\left(parent_{1}\left(\nu_{i}\right)+parent_{2}\left(\nu_{i}\right)\right)\times \left|parent_{1}\left(\nu_{i}\right)\right|}{\left|parent_{1}\left(\nu_{i}\right)+parent_{2}\left(\nu_{i}\right)\right|}$$

(6) $$child_{2}\left(\nu_{i}\right)=\frac{\left(parent_{1}\left(\nu_{i}\right)+parent_{2}\left(\nu_{i}\right)\right)\times \left|parent_{2}\left(\nu_{i}\right)\right|}{\left|parent_{1}\left(\nu_{i}\right)+parent_{2}\left(\nu_{i}\right)\right|}$$

Relative to PSO, this GPSO algorithm has a crossover operation that occurs after updating the position and velocity, and uses the gendered descendant particles rather than the parent particles for the next iteration. The crossover operation helps the descendant particles to inherit the advantages of their parent particles and maintains population diversity. The crossover mechanism selects the particle from all particles into the cross-matching pool with a certain degree of crossover probability, which has been determined beforehand and remains unchanged throughout the crossover process; matches any two particles in the pool randomly, determines the crossover point by the crossover weight *w_i_*, which has been calculated by the fitness value of particle, generates the descendant particle by the crossover operation.

### 2.2. Ratio of Mean to Variance Fitness Function

According to the purpose of change detection, we choose RMV as the fitness function for evaluating the fitness of particles in the GPSO algorithm, which denotes the availability of the candidate feature in the image object feature dataset.

In general, the mean and variance of a data set are related to the important feature information, so some features are used to compare the samples belonging to different classes [31]. This denotes the separability of a multi-class sample by normalizing the mean of the feature dataset according to its variance and comparing it among the different classes.

Assume that *A* and *B* are feature datasets belong to different classes, where *A* is the dataset of changed samples that have the feature *f*, and *B* is the dataset of unchanged samples that have the feature *f*. Then, the importance of feature *f* can be expressed by Equations (7) and (8):

(7) $$S_{f}=\frac{\left|mean_{f}\left(A\right)-mean_{f}\left(B\right)\right|}{V_{f}}$$

(8) $$V_{f}=\sqrt{\frac{Var_{f}\left(A\right)}{n_{A}}+\frac{Var_{f}\left(B\right)}{n_{B}}}$$

where *S_f_* is the significance of feature *f* and represents the potential to classify the two dataset *A* and *B*, *mean_f_* *(A)* and *mean_f_* *(B)* are the means datasets *A* and *B*, *Var_f_* (*A*) and *Var_f_* (*B*) are the variances of datasets *A* and *B*, and *n_A_* and *n_B_* are the number of samples in *A* and *B*, respectively.

The optimum features are selected from the feature dataset once the features have been sorted by the feature importance index*.* Assume that *M* features are to be selected, then the importance matrix *S* can be constructed by the obtained importance index of *M* features for each class, and the mean value of the feature importance *S_AVG_* can also be calculated using the feature importance matrix *S*, which has *M* feature importance indices:

(9) $$S_{AVG}=\frac{1}{M}\sum_{f=1}^{M}S_{f}$$

The objective function *J* is given as follow:

(10) $$J=V_{s}\times S_{AVG}^{2}$$

(11) $$V_{S}=\sum_{f=1}^{M}S_{f},\text{ if }S_{f}>S_{AVG}$$

It is apparent that larger values of *S_AVG_* and *J* indicate stronger classification capability of the selected feature subset from the feature dataset, so the fitness function of RMV is:

(12) $$Fitness_{\left(RMV\right)}=-V_{S}\times {\left(\frac{1}{M}\sum_{f=1}^{M}S_{f}\right)}^{2}$$

### 2.3. GPSO-Based Feature Selection for Object-Based Change Detection

**Figure 1:**
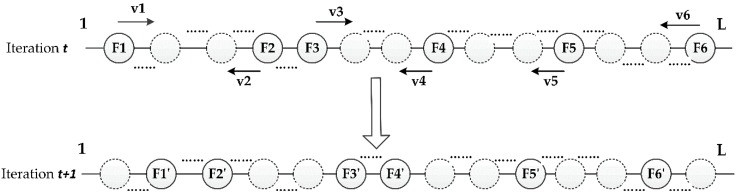
Illustration of one particle update for GPSO (Genetic Particle Swarm Optimization)-based feature selection.

When the proposed GPSO-based feature selection algorithm is used in the field of multiple features objected based change detection, the essential step is how the features are selected from the feature set. After the features have been extracted from the image object and the features set has been built in the field of OBCD, we give each feature an index, then these feature indices have been selected by GPSO-based feature selection algorithm. Figure 1 explains how the feature indexes update in the iteration of GPSO algorithm, and it illustrates one particle before and after one iteration step when selecting six features from the image object feature set which has *L* features. At the *t*-th iteration, six features are selected by each particle, x(*t*) = (*F1*,*F2*,*…*,*F6)^T^*, and GPSO determines the update v(*i*) = *(v1*,*v2*,*…*,*v6)^T^*. At the (*t* + 1)-th iteration, the selected feature indices becomes x(*t* + 1) = (*F1’*,*F2’*,*…*,*F6’)^T^.* According to the Figure 1, one particle *x_i_* denote a kind of feature combination, also can be regarded as a potential solution for the feature selection problem. At the end of iterations, the feature indices included in the best global solution *gbest_i_* of the particle swarm is the optimal result of feature selection.

**Figure 2:**
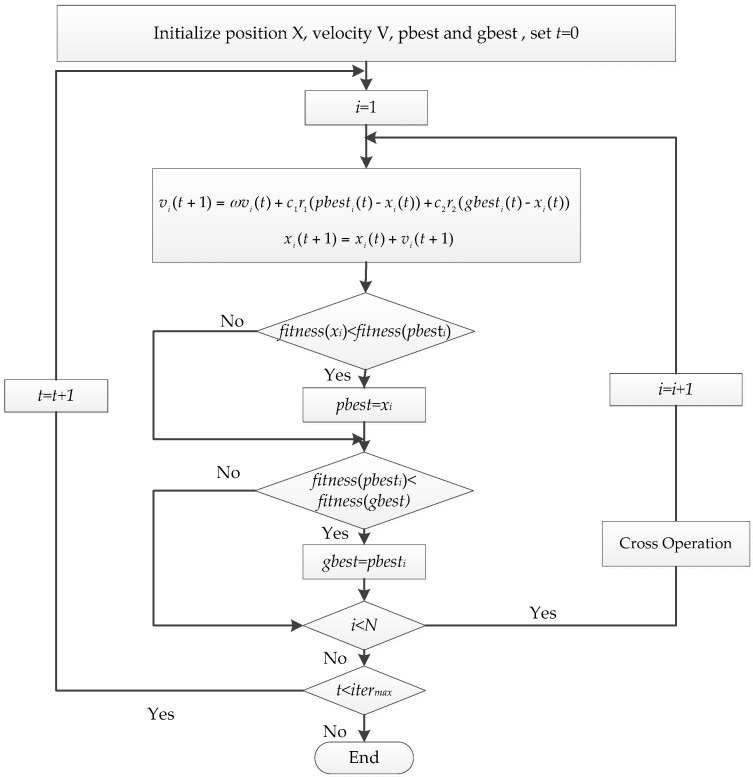
Flowchart of GPSO-based feature selection algorithm.

The procedures of the proposed GPSO-based feature selection algorithm are described as follows (Figure 2) [26].

- Step 1: Normalize and set the parameters, including the size of the particle swarm *N*, the learning factors *c_1_* and *c_2_*, the inertia weight factor *ω* and the maximum number of iterations *iter_max_*;
- Step 2: Assume that *M* features are about to be selected from the feature set, and randomly initialize *N* particles *x_i_*, and each particle includes *M* indices of the features to be selected;
- Step 3: Evaluate the fitness of each particle by Equation (12), and determine *pbest_i_* and *gbest_i_*;
- Step 4: Update the position and velocity vectors of each particle using Equations (1)–(6);
- Step 5: If the algorithm is converged, then stop; otherwise, go to Step 3 and continue;
- Step 6: The particle yielding the global optimum solution *gbest_i_* is the final solution and includes the selected feature subset.

Additionally, to validate the fitness convergence and OBCD accuracy of proposed GPSO-based feature selection algorithm, we choose Backtracking Search Optimization algorithm (BSO) and Cuckoo Search (CS) algorithm to compare with the GPSO algorithm.

BSO is also a kind of bionic algorithm and a population-based iterative evolution algorithm designed to be a global minimizer. The crossover strategy improved the global search ability of BSO, which is similar with the GPSO. Different with GPSO, BSO has a boundary control mechanism, which is effective in achieving population diversity, ensuring efficient searches, even in advanced generations [9], and it may be where more advanced than GPSO. Similar with the GPSO, CS algorithms also is a swarm intelligent algorithm, and the selection of optimal solution depends on the comparison of fitness value. Different with GPSO, CS updates the location and search path according to the random-walk mechanism, which has better global ability than GPSO and can keep a good balance between local search strategy and exploration of the entire search space. Nevertheless, the GPSO has improved its global search ability by importing into the cross operator in the iteration procedure.

## 3. Results

To validate the reliability and effectiveness of GPSO-based feature selection algorithm for multi-feature OBCD, two experiments were carried out on two pairs of Worldview-3 and Worldview-2 VHR remotely sensed images. Moreover, we also analyzed the fitness convergence and the accuracy of GPSO algorithm by comparison with BSO and CS algorithms.

### 3.1. Case A: Feature Selection for Multiple Features OBCD in Farmland Area

#### 3.1.1. Materials and Study Area

The multi-temporal VHR remotely sensed imagery data used in experiment Case A was taken by the Worldview-3 and Worldview-2 satellites [32]. The WV-3 VHR image was taken 17 October 2014, and the WV-2 VHR image was taken 27 September 2010. These two images have been cropped into sub-images of size 500 × 500 pixels with four spectral bands: blue, green, red and NIR, with spatial resolutions of 1.38 m and 1.84 m, respectively.

Geometric correction and relative radiometric correction of multi-temporal remotely sensed imagery are important procedures in VHR image change detection [33]. First, 50 ground control points are distributed across each image, ensuring that the root-mean-square error is less than one pixel through geometric calibration; Second, 50 pseudo-invariant feature points are selected, and the differences of solar radiation or atmospheric condition between the two images are eliminated or reduced by relative radiometric correction based on robust regression.

**Figure 3:**
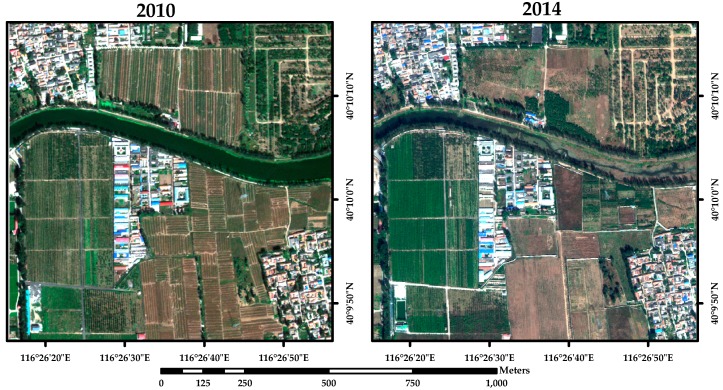
True color synthesis of images of study area nearby the Modern Agricultural Demonstrative Garden of Beijing (China) acquired by Worldview-2/3 VHR multispectral sensor on: (**a**) 27 September 2010; and (**b**) 20 October 2014.

Study area A is located to the north of Beijing (China), which is nearby the Modern Agricultural Demonstrative Garden of Beijing (Figure 3). It is typical farmland area, which dominates the changed land use type between 2010 and 2014. The changes in land cover include returning farmland to forest, and alterations to the texture or shape of farmland. To validate the accuracy of the test algorithms, 212 samples were collected in the study area, which were used to be the test samples.

#### 3.1.2. Image Object Feature Extraction

In the field of object-based image analysis, each image should be segmented into image objects, which are the basic unit of image analysis or processing. The image objects were obtained using the Multi-Resolution Segmentation (MRS) model [34,35] in this study. The parameters of the MRS model include the scale of segmentation, the weight of the shape criterion and the weight of the compactness criterion, which we set to be 150, 0.4 and 0.35, respectively, for analyzing the heterogeneity of images. These two multiple-feature temporal images were segmented into 526 objects using the MRS model, available in eCognition Developer 9.2 software (Trimble Navigation Ltd., Broomfield, CO, USA). To guarantee that corresponding objects at different times were segmented exactly the same way, we carried out image segmentation on eight image layers overlain on the two images with the four spectral bands.

Image object features consist of spectral, geometric and texture characteristics. Table 1 illustrates the feature set selected in this study, where the texture features are calculated using the grey level co-occurrence matrix (GLCM) [36,37]. There are 20 kinds of image object features in the feature set, and these feature indices were coded in a certain order.

In OBCD, change information is usually reflected in the variance image, which is obtained by directly comparison or image transformation methods [38]. The changed objects then can be extracted from the variance image by threshold segmentation. Considering the purpose of this study, we choose the multivariate alternative detection algorithm [39] to build the variance image, as it is well suited to multi-feature OBCD, and choose the histogram curvature analysis algorithm to extract the changed objects. The resulting OBCD model is the OB-HMAD (Objected-Based Hybrid Multivariate Alternative Detection) algorithm [40], which has the advantages of multi-tunnel processing and maximum retention of original change information, and it can protect the diversity between multi features in OBCD and obtain variation image with the enhance change information by nonlinear transformation, so it is suitable for the OBCD method in this study.

#### 3.1.3. Convergence Analysis of GPSO

The convergence analysis of GPSO-based feature selection algorithms is related to the global search ability and efficiency of the algorithm, and compares the average fitness and the optimum fitness. The average fitness (*fitness_avg_*) represents the efficiency of the algorithm, and can represent the global search ability in combination with the optimum fitness (*fitness_opt_*).

The initial parameters in the GPSO-based feature selection algorithm are set as follows: the initial size of the particle swarm is *N* = 60, the learning factors are *c*_1_ = 2.8 and *c*_2_ = 1.3, the inertia weight factor is *ω* = 0.9, the maximum number of iterations is *iter_max_* = 80, and the number of features to be selected is *M* = 6, so each particle includes six types of feature. To analyze the fitness convergence of the GPSO-based feature selection algorithm, we compare the performance with the BSO and CS algorithms. Additionally, our GPSO, BSO and CS algorithms have been developed and implemented in Matlab 2010b software.

**Figure 4:**
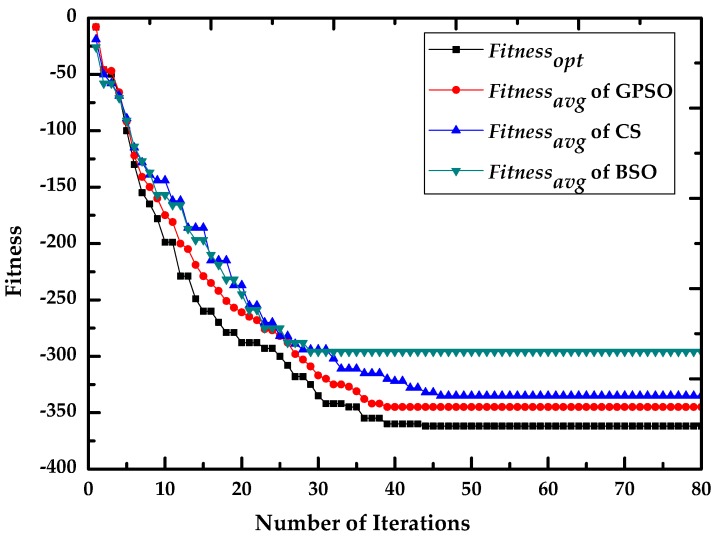
Comparison analysis of fitness convergence with GPSO, CS and BSO in Case A.

In Table 2, *Iteration_con_* is the number of iterations required for the algorithm to converge, and *D_con_* is the difference between the final values of *fitness_avg_* and *fitness_opt_*. As indicated in Table 2 and Figure 4, BSO has the fastest convergence speed, but *D_con_* for BSO is much larger than in the other cases, which means that BSO tends to convergence locally. Conversely, CS escapes local optima but has a slower convergence speed than GPSO. The optimum final value of *fitness_avg_* is obtained by GPSO and the convergence curve are more stable than in the others. Additionally, the parameters of *D_con_* for GPSO in different cases are close to each other. The result reveals that GPSO is superior at finding optima avoiding premature convergence compared with CS and BSO.

#### 3.1.4. Accuracy Evaluation of Change Detection Based on OB-HMAD

To validate the applicability of the proposed GPSO-based feature selection algorithm in OBCD for VHR remotely sensed imagery, the image object features to be selected by the algorithm are processed by the OB-HMAD model [41]. The error confusion matrix for OBCA is then constructed from the test samples by comparison of the result image and ground truth data, which has the change trajectory defined by the field investigation and visual judgment from Google Earth and actual terrain classification image. This ground truth data can be recognized as the reference data to compare the results of change detection with different feature selection algorithm in OBCD. The OBCD accuracy can be evaluated using the false negative rate (probability of missing detection), the false positive rate (probability of false detection), the overall accuracy (probability of correct detection), and the Kappa coefficient calculated from the error confusion matrix [40].

Table 3 shows the results of feature selection based on BSO, CS and GPSO, with feature selection results displayed as 20-bit binary code, all GLCM-based features have four directional values (0°, 45°, 90° and 135°).

**Figure 5:**
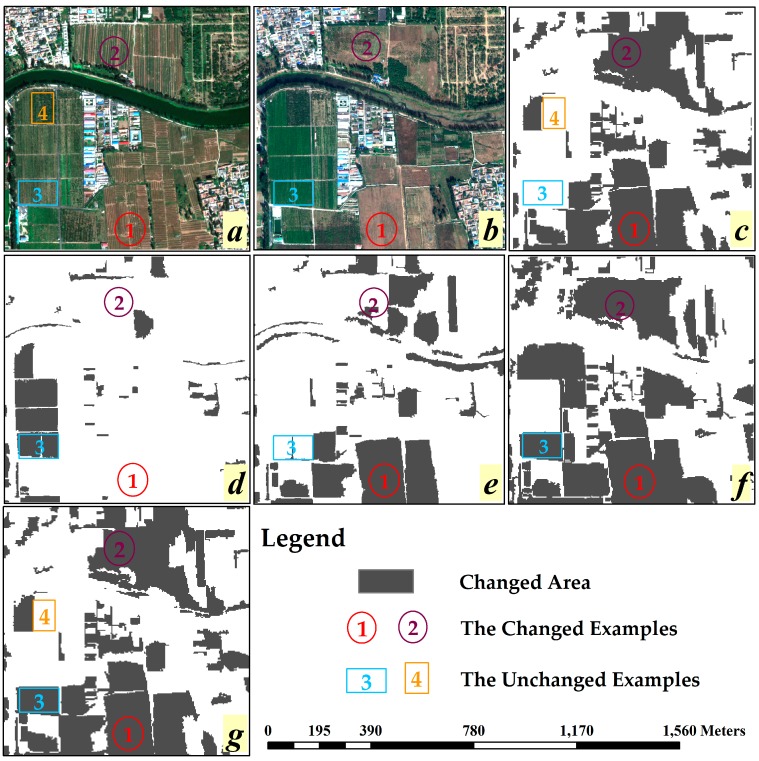
Change detection results based on OB-HMAD(Objected Based-Hybrid Multivariate Alternative Detection) with different feature selection algorithms in Case A: (**a**) Worldview-2 VHR(Very High Resolution) remotely sensed image from 2010; (**b**) Worldview-3 VHR remotely sensed image from 2014; (**c**) the reference map obtained by ground truth data; (**d**) OBCD(Objected Based Change Detection) result with a single spectral feature (average of bands); (**e**) OBCD result with multiple features selected by BSO(Backtracking Search Optimization ); (**f**) OBCD result with multiple features selected by CS(Cuckoo Search); and (**g**) OBCD result with multiple features selected by GPSO.

Figure 5 illustrates the OB-HMAD results of Case A for single and multiple features selected by the BSO, CS and GPSO algorithms, where Figure 5a,b shows the original images, Figure 5c is the reference map obtained by the ground truth data, Figure 5d shows the OBCD result with a single spectral feature (average of bands), and Figure 5e–g shows the OBCD results with multiple features. Figure 5d is significantly different from Figure 5c as well as the other subfigures, containing several false negative errors and false positive errors. To analyze the problems of missing detection and false detection, four examples are shown in Figure 5. According to the reference data, changed areas 1 and 2 relate to the missing detection problem, and unchanged areas 3 and 4 relate to the false detection problem.

Area 1, which has been marked as changed area in Figure 5c, was covered by wheat in 2010, and it has many obvious ridges, but the land cover type has changed by 2014 and the ridges have disappeared. The texture of the image objects covering this area have changed so it has been marked *changed* in Figure 5e–g, but this area is not distinguished in Figure 5d. Similar to area 1, the direction of the texture in the image objects covering area *2* has changed, but it still fails to be distinguished in Figure 5d,e. This is possibly related to a lack of GLCM-contrast (90°) in the feature set selected by the BSO algorithm. In area 3, which has been marked as unchanged area in Figure 5c, the spectral feature has changed so it is marked as changed in Figure 5d, but area 3 is actually an unchanged area. Referring to other features, the multi feature OBCD methods give accurate results as shown in Figure 5e–g*.* In particular, area 4 is mistakenly identified as changed area in Figure 5e but is correctly identified in Figure 5d, suggesting that the mutual interference of multiple features may affect the accuracy of OBCD. Therefore, while multi feature OBCD algorithms can avoid many missing and false detection problems, this method may be a reliable reference but should not be used as the only criterion.

Table 4 shows the accuracy evaluation results for these algorithms in these two experiment cases, and the data in Table 4 are computed by the confusion matrix, which is obtained by comparing test sample points in change detection result image and the ground truth image respectively. In Case A, it is obvious that the precision of multi feature OBCD methods is better than that of single-feature methods. The CS algorithm has more problems with false detection (FPR_CS_ = 36.49%) and BSO has more problems with missing detection (FNR_BSO_ = 54.35%), but GPSO has the highest value of the accuracy evaluation indices (OA_GPSO_ = 84.17%).

### 3.2. Case B: Feature Selection for Multiple Features OBCD in Urban Area

#### 3.2.1. Materials and Study Area

The Case B data are made up of a pair of WV-2 VHR images taken on 12 September 2012 and 20 September 2013, and they have also been cropped into sub-images of size 1000 × 1000 pixels with four bands. During data preprocessing, the similar relative radiometric and geometric corrections with Case A were carried out to make the two images as comparable as possible.

**Figure 6:**
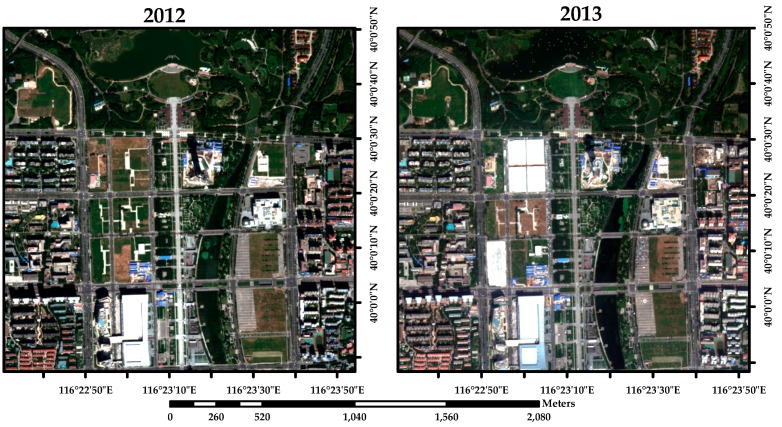
True color composition of images of study area nearby the Olympic Park of Beijing (China) acquired by Worldview-2 VHR fusion image on: (**a**) 12 September 2012; and (**b**) 20 September 2013.

The study area B is located to the heart of Beijing, which is around the Beijing Olympic Park. As the most important park with multiple eco-system service function, the dynamic change of the around buildings have some significantly effect on the park. The dominating changed land cover is the change of construction, where the shadow of high buildings caused the mistake of change detection based on spectral feature of images. To validate the proposed algorithms, 228 samples were used for accuracy assessment. Figure 6 shows the pairs of VHR remotely sensed images.

#### 3.2.2. Image Object Feature Extraction

Based on the MRS model, the image was segmented into 837 objects in eCognition Developer 9.2 software. First, the two images were overlaid to one image with eight image layers; then the image layer weights in Multi-Resolution Segmentation model are all set to “1” for each image layer. This procedure of image overlay can guarantee that the two images have the same edge of corresponding objects. In this study case, 20 kinds of image object features were extracted to build the feature set, such as average of bands, NDVI, shape index, density, GLCM-correlation, GLCM-contrast, GLCM-ang.2nd moment and GLCM-Homogeneity, and these feature indices were coded in a certain order.

#### 3.2.3. Convergence Analysis of GPSO

The initial parameters in the GPSO-based feature selection algorithm are set as follows: the initial size of the particle swarm *N* = 80, the learning factors *c*_1_ = 2.6 and *c*_2_ = 1.5, the inertia weight factor *ω* = 0.9, the maximum number of iterations *iter_max_* = 100, and the number of features to be selected *M* = 7, so each particle includes seven types of feature. Similar with Case A, we also chose the CS and BSO algorithms to analyze the fitness convergence of algorithms, relative to the GPSO-based feature selection algorithm.

**Figure 7:**
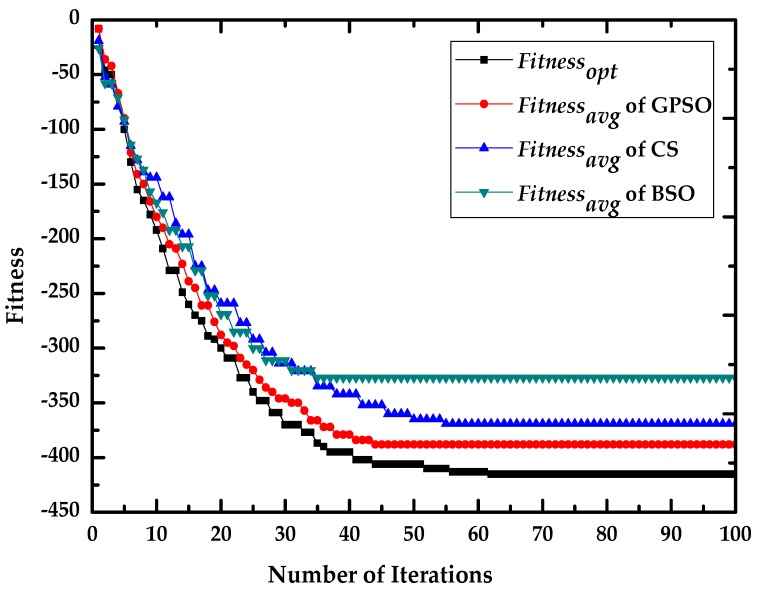
Comparison analysis of fitness convergence with BSO, CS and GPSO in Case B.

As indicated in Table 5 and Figure 7, the two groups of experiment results are similar: BSO has the fastest convergence speed and the largest *D_con_*, indicating that BSO tends to convergence locally. Moreover, CS has the best global search ability but has a slower convergence speed than GPSO. The optimum final value of *fitness_avg_* is obtained by GPSO and the convergence curve are more stable than in the others. Additionally, the parameters of *D_con_* for GPSO in different cases are close to each other. It proved again that GPSO has better ability to avoid the avoiding premature convergence compared with CS and BSO.

#### 3.2.4. Accuracy Evaluation of Change Detection Based on OB-HMAD

Based on the OB-HMAD algorithm, the change results have been obtained with the features selected by GPSO-based feature selection algorithm. The reference data were defined by the field investigation and visual judgment from Google Earth image. Then, we also use FNR, FPR, OA and Kappa coefficient, calculated by the error confusion matrix, to evaluate the accuracy of change detection results based on these feature selection algorithms in OBCD.

Table 6 shows the results of feature selection based on GPSO, CS and BSO, and these algorithms have selected seven features. According to this table, the selected features are similar with each other among these algorithms, and there are four kinds of features all selected by these algorithms.

**Figure 8:**
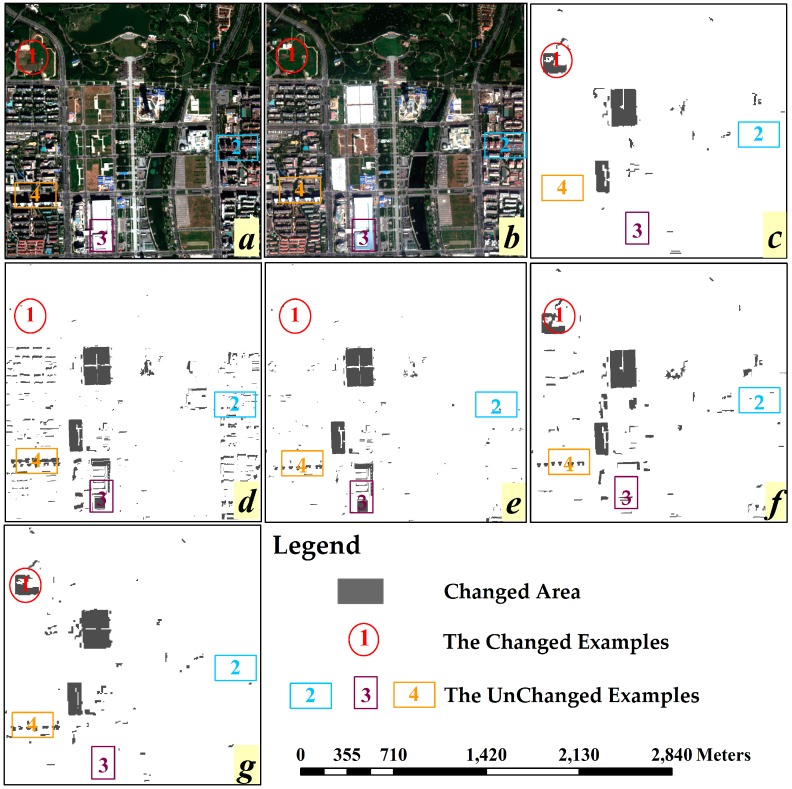
Change detection results based on OB-HMAD with different feature selection algorithms in Case B: (**a**) Worldview-2 VHR remotely sensed image from 2012; (**b**) Worldview-2 VHR remotely sensed image from 2013; (**c**) the reference map obtained by ground truth data; (**d**) OBCD result with a single spectral feature (average of bands); (**e**) OBCD result with multiple features selected by BSO; (**f**) OBCD result with multiple features selected by CS; and (**g**) OBCD result with multiple features selected by GPSO.

Similar with the results analysis of Case A, the results of Case B have also been divided into several subfigures, as shown t in Figure 8. By analyzing the four example area in Figure 8, we can obtain similar results with Case A: OBCD with single spectral feature has poorer performance than ones with multi features, and Figure 8g is close to the Figure 8c, which means the OB-HMAD result with multiple features selected by GPSO is the closet to the real situation. Besides, Figure 8d,e has some missing detection problems in terms of area 1, while Figure 8e–f has some false detection problems in terms of area 3, these mistakes, caused by different illumination angle, can be corrected with the assistance of GLCM-Homogeneity. The false detection caused by the shadow of a high building can also be avoided with the assistance of texture features, as can be seen in area 2. In particular, area 4 is a pseudo-changed area and this pseudo change is caused by the difference of shooting angle of sensor. This area has been mistakenly identified as changed area by these algorithms but has not changed.

Table 7 shows the accuracy evaluation results for these algorithms in this experiment case; it is validated that single-feature OBCD could not detect enough kinds of change and had poor accuracy of OBCD. The BSO algorithm has more problems with false detection (FPR_CS_ = 16.72%) and missing detection (FNR_BSO_ = 27.23%), and CS and GPSO have a similar result, but GPSO is superior at the accuracy evaluation indices (OA_GPSO_ = 83.59%). It is worth noting that there are some pseudo or slightly changed objects in the context of a complex urban environment, but these algorithms mistakenly regarded these pseudo changed objects as changed objects.

## 4. Discussion

Some parameters affect the accuracy and efficiency of the GPSO-based feature selection algorithm used in OBCD for VHR remotely sensed imagery, so we should analyze the sensitivity of the three algorithms. In this study, we choose the images of Case A as the experiment subject to analyze the sensitivity of the algorithms, and the sensitivity analysis of the GPSO-based feature selection algorithm focuses on the influence of the number of features to be selected and the size of the particle swarm. The RMV fitness function is also analyzed to compare with other fitness functions.

### 4.1. Number of Features to Be Selected

As one of the initial parameters of the GPSO-based feature selection algorithm, the number of features to be selected, *M*, affects the efficiency and reliability of the algorithm. A larger value of *M* creates more data redundancy and increases the running time of the algorithm, while a smaller value of *M* decreases the accuracy and loses more critical change information. Thus, it is necessary to analyze the sensitivity of the accuracy of OBCD to *M* to find the optimum value of *M*.

**Figure 9:**
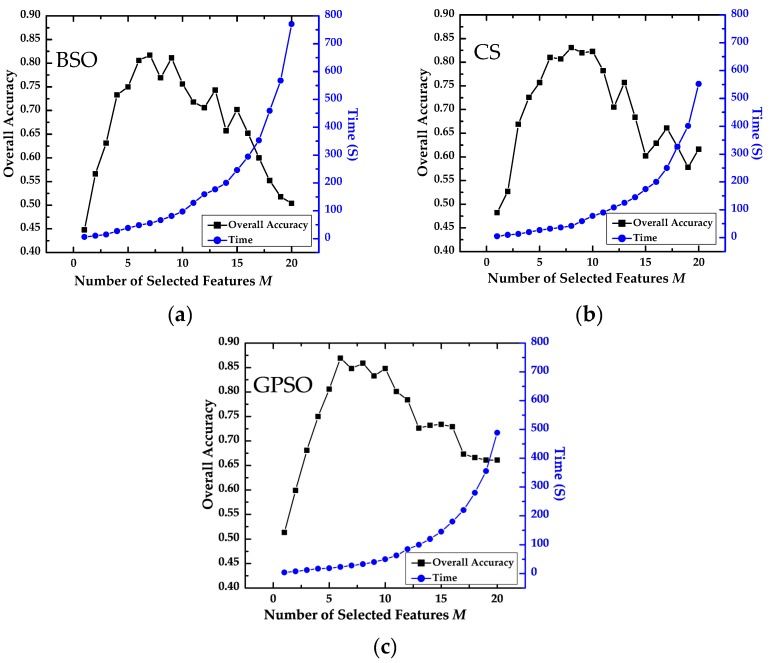
Impact of the number of features on precision and running time of OBCD with different feature selection algorithms: (**a**) BSO algorithm; (**b**) CS algorithm; and (**c**) GPSO algorithm.

Figure 9 illustrates the sensitivity analysis results for the influence of *M* on the overall accuracy and running time of our algorithm for OBCD. The overall accuracy is calculated from the error confusion matrix constructed from the test samples, and the running time for the GPSO and OB-HMAD algorithms on a personal computer with an Intel Core i7 2.93-GHZ CPU and 4 GB of memory is shown on the secondary Y-axis. The other parameters of GPSO are as follows: the initial number of particles is *N* = 60, the learning factors are *c*_1_ = 2.8 and *c*_2_ = 1.3, the inertia weight factor is *ω* = 0.9, and the maximum number of iterations is *iter_max_* = 100.

In Figure 9 and Table 8, the overall accuracy of the algorithm increases steadily as *M* increases from 1 to 9; however, when *M* ≥ 10, the accuracy decreases. Increasing *M* drives an increase in the running time, particularly when *M* ≥ 10. Taking into consideration the accuracy and efficiency of the algorithm, the optimum number of features to select is six to eight. Compared with CS and BSO, GPSO has the best performance in terms of accuracy, as the maximum and mean values for GPSO are higher than in the other cases, and the standard deviation and average change rate are both lower than in the other cases. This means that GPSO is less sensitive to the number of features to be selected and is less susceptible to the influence of the initial value.

### 4.2. Size of the Particle Swarm

It is essential to discuss the effect of the size of the particle swarm on the fitness convergence and the running time of algorithm, as a small particle swarm may lead to local convergence and a large particle swarm increases the running time of the algorithm. Trelea found that a suitable size for the particle swarm is between 20 and 100 [42]. In our experiment, we choose *N* = 20, 40, 60, 80 and 100 as the initial numbers of particles, denoted by 20GPSO, 40GPSO, 60GPSO, 80GPSO and 100GPSO, respectively. The number of features to be selected is *M* = 6, the learning factors are *c*_1_ = 2.8 and *c*_2_ = 1.3, the inertia weight factor is *ω* = 0.9, and the maximum number of iterations is *iter_max_* = 100.

**Figure 10:**
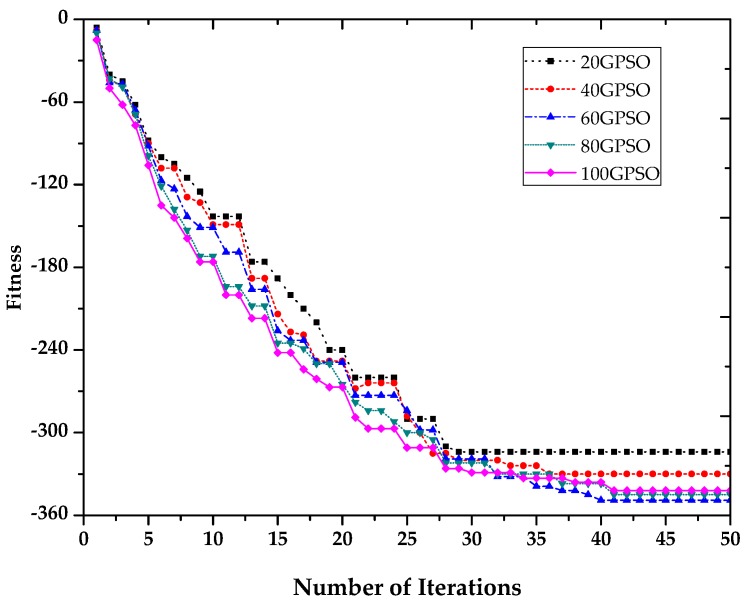
Fitness convergence curves of GPSO-RMV based on different scales of particle swarm.

In Figure 10 and Table 9, the convergence speed and running time of the algorithms increase as *N* increases, but the final converged fitness values, which represents the error rate of the algorithm, are similar, except in the case of the 20GPSO algorithm. While the algorithm converges faster in this instance, the final converged fitness value is higher, meaning that 20GPSO has a problem with premature convergence. Overall, 60GPSO has the minimum converged fitness value, so we consider that GPSO with *N* = 60 has the best performance in terms of precision and running time.

### 4.3. Comparison of Different Fitness Functions

Because the fitness function determines the applicability of the algorithm, selection of the fitness function for the GPSO-based feature selection algorithm should be in accordance with the purposes of our research.

To analyze the applicability of RMV, we choose Jeffreys–Matusita Distance (JMD) [43] and Nearest Neighbor Classifier (NNC) to compare with RMV for the fitness convergence of the algorithms. Based on these fitness functions, the GPSO-based feature selection algorithms are denoted by GPSO-RMV, GPSO-NNC and GPSO-JMD, respectively.

**Figure 11:**
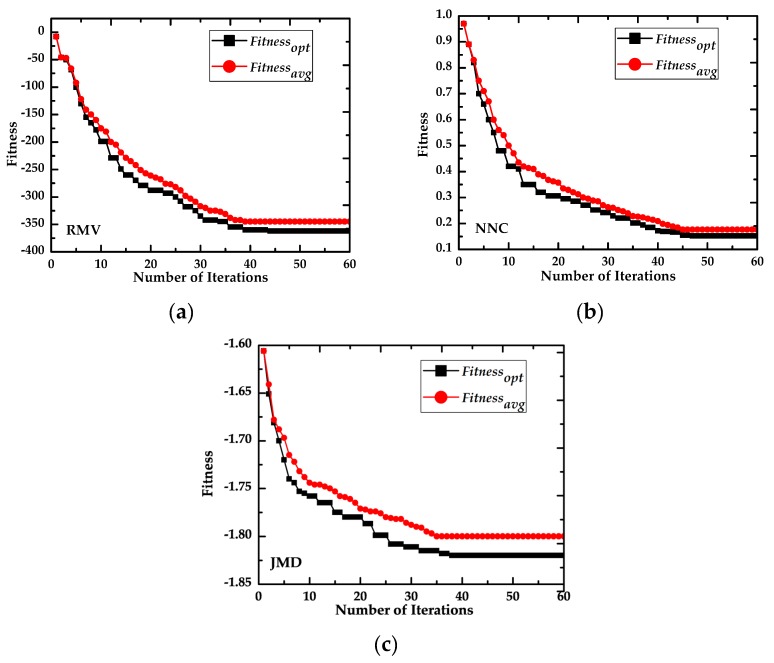
Comparison of fitness convergence with different fitness functions: (**a**) RMV (Ratio of Mean to Variance); (**b**) NNC (Nearest Neighbor Cassifier); and (**c**) JMD (Jeffreys–Matusita Distance).

Figure 11 shows the fitness convergence curve for the three fitness functions, all of which converge within 60 iterations. Note that the *fitness_avg_* curves are also close to the corresponding *fitness_opt_* curve. During the first 20 iterations, convergence is fast and there is only a small gap between *fitness_avg_* and *fitness_opt_*. The convergence of GPSO-JMD is faster than the other two algorithms, with convergence of *fitness_avg_* and *fitness_opt_* occurring after 35 and 38 iterations, respectively, but there is a big gap between the final values of *fitness_avg_* and *fitness_opt_*, meaning that GPSO-JMD may have some problems with premature convergence. For the GPSO-NNC algorithm, the *fitness_avg_* and *fitness_opt_* curves are very close so it has the lowest error rate, but the *fitness_avg_* and *fitness_opt_* values do not converge until after around 50 iterations. For the GPSO-RMV algorithm, the variance between the average and optimum fitness is small and they converge after the same number of iterations. Overall, the values for GPSO-RMV show close to global convergence, which means that it has powerful global search ability.

## 5. Conclusions

This study applied GPSO to select the optimum image object features for OBCD of VHR remotely sensed images and chose RMV as the fitness function. We analyzed the fitness convergence and accuracy of OBCD in the GPSO-based feature selection algorithm, and discussed the influence of the number of features to be selected and the size of the particle swarm on the precision and efficiency of the algorithm. Additionally, we analyzed the adaptability of the RMV fitness function and compared it with two other fitness functions, JMD and NNC.

GPSO has the advantages of strong global search ability, high efficiency and stability, and can effectively avoid premature convergence. The experiments prove that the GPSO-based feature selection algorithm performs better than other algorithms in OBCD of VHR remotely sensed images. In the sensitivity analysis of the GPSO-based feature selection algorithm, a larger the number of features to be selected increases the precision and the computational cost of the algorithm when the number of features to be selected is less than 10. The experiments show that the algorithm has high precision and is fast if the number of features is between six and eight. Additionally, the experiments also found that GPSO is not affected as much by the number of features to be selected as the other two algorithms with which it was compared. Similarly, the size of the particle swarm also affects the convergence speed of the algorithms, with the optimum number of initial particle determined to be 60.

As the discriminatory criterion for the GPSO-based feature selection algorithm, the RMV fitness function was analyzed and compared with the JMD and NNC functions. The experiments show that the fitness convergence speed of three fitness functions are similar, and that their final converged fitness values are all close to the optimum fitness value. Relatively speaking, RMV is more suitable to be the fitness function of GPSO-based feature selection algorithm because of the convergence speed and precision of the algorithm.

Meanwhile, the OBCD experiment based on OB-HMAD also showed that multi-feature change detection has higher precision than single-feature change detection, and that multi-feature change detection can distinguish some areas where texture or shape has changed, which is not possible with single-feature change detection. Additionally, the experiment also exposed some problems caused by mutual interference between the features. This means that the GPSO-based feature selection algorithm requires artificial visual interpretation to assist in OBCD.

## Figures and Tables

**Table 1:** Spectral, texture and geometric features of image objects.

Case	Spectral	Geometric	Texture (GLCM-Based)
Case A	Mean	Shape Index	GLCM-Correlation
	NDVI	Density	GLCM-Contrast
			GLCM-Entropy
			GLCM-Mean

**Table 2:** Analysis results of fitness convergence in Case A.

Case	Feature Selection Algorithms	BSO	CS	GPSO
Case A	*Iteration_con_*	29	46	40
	*Final fitness_avg_*	−296	−335	−345
	*D_con_*	64	27	17

**Table 3:** Results of feature selection with different algorithms in Case A.

Case	Algorithms	Selected Features
Case A	BSO	Mean, NDVI, GLCM-Correlation (0°, 90°), GLCM-Contrast (0°), GLCM-Mean (0°)
	CS	Mean, NDVI, GLCM-Correlation (0°), GLCM-Contrast (0°, 90°),GLCM-Entropy (0°)
	GPSO	Mean, NDVI, GLCM-Correlation (0°), GLCM-Contrast (0°, 90°), GLCM-Entropy (0°)

**Table 4:** Accuracy evaluation of OBCD with different feature selection algorithms in Case A.

Case	Methods	FNR (%)	FPR (%)	OA (%)	Kappa
Case A	Single-Feature	69.56	32.43	53.33	0.0648
	BSO	54.35	14.87	70.06	0.3267
	CS	6.52	36.49	75.00	0.5187
	GPSO	10.87	18.92	84.17	0.6771

FNR, false negative rate; FPR, false positive rate; OA, overall accuracy.

**Table 5:** Analysis results of fitness convergence in Case B.

Case	Feature Selection Algorithms	BSO	CS	GPSO
Case B	*Iteration_con_*	35	55	44
	*Final fitness_avg_*	−327	−369	−388
	*D_con_*	88	46	27

**Table 6:** Results of feature selection with different algorithms in Case B.

Case	Algorithms	Selected Features
Case B	BSO	Mean, NDVI, GLCM-Correlation (90°), GLCM-Contrast (0°, 90°), GLCM-2nd Angust moment (0°), GLCM-Homogeneity (0°)
	CS	Mean, GLCM-Correlation (0°, 90°), GLCM-Contrast (0°, 90°), GLCM-2nd Angust momen t (0°, 45°)
	GPSO	Mean, GLCM-Correlation (0°, 90°), GLCM-Contrast (0°), GLCM-2nd Angust moment (0°, 45°), GLCM-Homogeneity (0°)

**Table 7:** Accuracy evaluation of OBCD with different feature selection algorithms in Case B.

Case	Methods	FNR (%)	FPR (%)	OA (%)	Kappa
Case B	Single-Feature	32.35	57.45	49.22	0.0726
	BSO	16.47	27.23	72.63	0.4517
	CS	11.76	22.47	79.91	0.5712
	GPSO	8.82	19.16	83.59	0.6314

**Table 8:** Accuracy statistics of sensitive analysis of *M* in OBCD.

Algorithms	Maximum	Mean	Standard Deviation	Average Change Rate
BSO	81.7%	67.0%	10.7%	8.1%
CS	83.1%	69.4%	10.2%	7.8%
GPSO	86.9%	73.1%	9.1%	5.3%

**Table 9:** Sensitivity analysis results of size of particle swarm.

Algorithm	*Iteration_con_*	*Final Fitness_avg_*	*Running Time* (s)
20GPSO	25	−314	30
40GPSO	36	−330	45
60GPSO	40	−345	70
80GPSO	44	−340	125
100GPSO	44	−332	150
